# The role of bile acid-activated receptor TGR5 in inflammation and liver diseases

**DOI:** 10.3389/fphys.2026.1747341

**Published:** 2026-02-17

**Authors:** Hongyan Xiang, Huanyu Xiang, Shuyun Wang, Peiyu Wu, Zhidan Luo, Jie Zhang

**Affiliations:** 1 Department of Geriatrics, Chongqing General Hospital, Chongqing University, Chongqing, China; 2 Department of VIP, Chongqing General Hospital, Chongqing University, Chongqing, China

**Keywords:** bile acids, hepatocellular carcinoma, inflammation, liver fibrosis, metabolic dysfunction-associated fatty liver disease, tgr5

## Abstract

Takeda G-protein-coupled receptor 5 (TGR5), a bile acid receptor, has been recognized as an important signal molecule with roles extending far beyond bile acid homeostasis. Its activation has been shown to ameliorate metabolic disorders and suppress inflammatory responses through diverse mechanisms. Expressed widely in both parenchymal and non-parenchymal cells of the liver, TGR5 plays a central role in hepatic physiology and disease. This review consolidates current evidence on the involvement of TGR5 in various liver pathologies, including metabolic dysfunction-associated steatohepatitis, cholestatic diseases, liver fibrosis, and hepatocellular carcinoma. Additionally, we summarize the regulatory functions of TGR5 in immune cells and inflammatory signaling pathways. We emphasize TGR5 as a promising therapeutic target for a range of chronic liver diseases, given its pivotal role in modulating inflammation and metabolism. Future research should focus on developing tissue-specific TGR5 agonists to enhance therapeutic efficacy and reduce systemic side effects, as well as elucidating its context-dependent dual roles in hepatocarcinogenesis to ensure safe clinical application.

## 1 Introduction

Takeda G-protein-coupled receptor 5 (TGR5), also known as G protein-coupled bile acid receptor 1 (GPBAR1), GPCR19, GPR131, or membrane-type receptor for bile acids (MBAR) is a member of the G protein-coupled receptor (GPCR) family. It was first identified as a bile acid (BA) receptor in the early 2000s (Maruyama et al., 2002; Kawamata et al., 2003). This discovery marked a pivotal advance by revealing that BAs function not only as digestive surfactants but also as potent signaling molecules. Over the past 2 decades, TGR5 has been established as protective regulator of metabolic and inflammatory pathways, showing therapeutic promise for a spectrum of conditions, including liver diseases, diabetes, cardiovascular disorders, and even psychiatric illnesses (Darmanto et al., 2025; Guan et al., 2022; Jin et al., 2024).

Among the recognized BA-sensing receptors, the membrane-bound TGR5 and the nuclear receptor farnesoid X receptor (FXR) represent the two most extensively studied prototypes (Thibaut and Bindels, 2022). Their distinct physiological roles are reflected in different ligand specificity: FXR is preferentially activated by primary BAs such as chenodeoxycholic acid (CDCA) and cholic acid (CA), whereas TGR5 exhibits higher affinity for secondary BAs, such as lithocholic acid (LCA) and deoxycholic acid (DCA), which are generated by gut microbiota (Mulak, 2021; Xia et al., 2022). The activation of TGR5, which occurs with a potency order of LCA > DCA > CDCA > CA (Alenezi et al., 2023), typically elicits Gαs coupling, leading to the stimulation of the cAMP-protein kinase A (PKA) pathway. This canonical signaling cascade underpins TGR5’s roles in regulating proliferation, metabolism, and inflammation (Gou et al., 2023). Interestingly, beyond the predominant Gαs pathway, TGR5 has also been documented to couple with Gαi protein in nonciliated cholangiocytes, resulting in the inhibition of cell proliferation (Masyuk et al., 2013). However, whether this alternative Gαi coupling represents a broader mechanism in other cell types remains an open question, highlighting an area for future investigation.

TGR5 is ubiquitously expressed across multiple organs, including the heart, brain, kidney, and gastrointestinal tract (Wang et al., 2016; Wang et al., 2024; Li et al., 2024b; Thibaut and Bindels, 2022). In the liver, TGR5 is predominantly localized in non-parenchymal cells, such as liver sinusoidal endothelial cells (LSECs), activated hepatic stellate cells (HSCs), intrahepatic and extrahepatic cholangiocytes, and Kupffer cells (KCs) (Zeng et al., 2023), though it is also detectable in parenchymal hepatocytes (Keitel and Häussinger, 2012; Holter et al., 2020; Deutschmann et al., 2018; Bidault-Jourdainne et al., 2021). This cell-specific distribution implies its multi-faceted roles in modulating inflammation, metabolism, and fibrosis in the liver. Given that inflammation is a central driver in the pathogenesis of many chronic liver conditions, this review will focus on the anti-inflammatory effects mediated by TGR5 in immune cells and discuss their relevance to the pathogenesis and potential treatment of liver diseases, including metabolic dysfunction-associated steatohepatitis (MASH), cholestatic disorders, liver fibrosis/cirrhosis, and hepatocellular carcinoma (HCC).

## 2 TGR5 and inflammation

The anti-inflammatory property of TGR5 was first discovered in monocytes and macrophages. Researchers observed that BAs could reduce the phagocytic activity of monocytes and macrophages by suppressing lipopolysaccharide (LPS)-induced pro-inflammatory cytokines including interleukin (IL)-1, IL-6, and tumor necrosis factor-α (TNF-α) (Calmus et al., 1992; Greve et al., 1989). Kawamata et al. (2003) further revealed that this anti-inflammatory effect was mediated by BA membrane receptor TGR5 (Kawamata et al., 2003). Further studies have established that TGR5 is highly expressed across a broad range of innate immune cells, including dendritic cells, NK cells, and NKT cells (Shi et al., 2025). Beyond the innate immune system, emerging evidence indicates that TGR5 also directly modulates adaptive immune responses by acting on T cells (Li et al., 2023; Zheng et al., 2024), although the underlying mechanisms require further elucidation. Collectively, the widespread expression and functional involvement of TGR5 in both innate and adaptive immunity underscore its role as a pivotal global immune regulator. The specific regulatory effects of TGR5 across different immune cell types are summarized in Table 1.

**TABLE 1:** The regulatory role of TGR5 in different immune cells.

Cell type	Regulation effects
Macrophages	Inhibition of NF-κB activity and proinflammatory cytokine production (Pols et al., 2011); Inhibition macrophage infiltration and proinflammatory M1 polarization (Zhou et al., 2021); inhibited NLRP3-mediated M1 macrophage polarization (Shi et al., 2021)
Dendritic cells	Downregulation of the NF-κB and MAPK pathways, leading to apoptosis and autophagy in Dendritic cells (Hu et al., 2022; Hu et al., 2021)
NK cells	Suppressing IFN-γ and TNF-α and reducing NK cell cytotoxicity (Wei et al., 2025)
NKT cells	Promoting the NKT cells polarization toward a NKT10, a regulatory, IL-10 secreting cell subset (Biagioli et al., 2019)
CD4+T	Promoting differentiation of CD4^+^T cells to the proinflammatory Th17 subset (Li et al., 2023)
CD8+T	Activation of CD8^+^ T cell via mTOR/OXPHOS signaling (Zheng et al., 2024)

**FIGURE 1:**
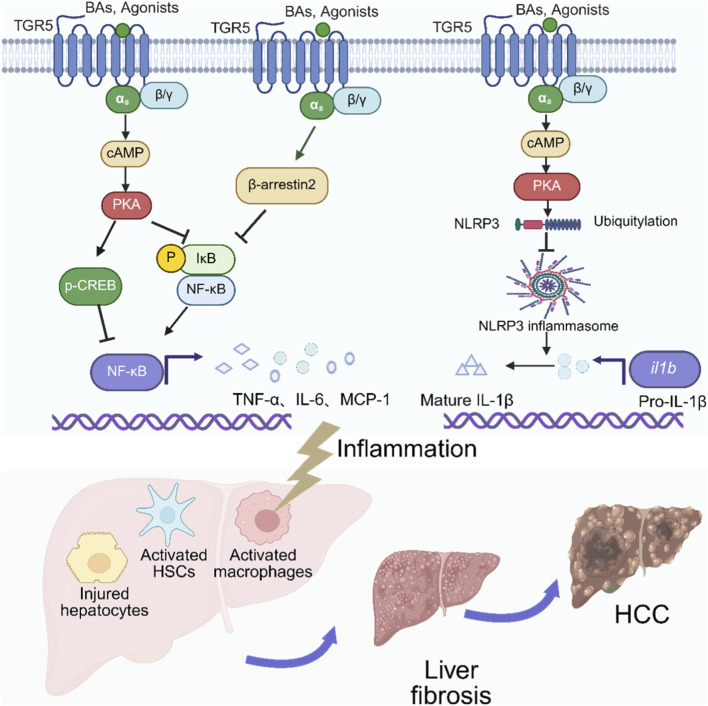
TGR5-mediated anti-inflammatory mechanisms and their implications in chronic liver disease. BAs, bile acids; cAMP, cyclic AMP; PKA, Protein kinase A; p-CREB, Phosphorylated cAMP response element-binding protein; NF-κB, nuclear factor kappa- B; IκB, inhibitor a of NF- κB; NLRP3, NOD-like receptors family pyrin domain containing three; il1b, Interleukin-1β. HSCs, Hepatic stellate cells; HCC, hepatocellular carcinoma. The figure was created in https://BioRender.com.

TGR5 has been reported to suppress the classical NF-κB signaling pathway through multiple mechanisms through multiple mechanisms (Figure 1). One primary mechanism involves the enhancement of the IκBα/β-arrestin2 interaction, which stabilizes IκBα and prevents NF-κB activation (Xiao et al., 2020; Wang et al., 2011). As evidenced by β-arrestin2 knockdown experiments, this interaction is indispensable for the suppressive effect of TGR5 on NF-κB transcriptional activity and its target gene expression (Wang et al., 2011). Other significant pathways are mediated by the cAMP-PKA axis. On one hand, this axis directly inhibits IκBα phosphorylation, thus inhibiting subsequent p65 nuclear translocation (Hu et al., 2021; Zhao et al., 2023). On the other hand, it promotes cAMP response element-binding protein (CREB) phosphorylation, which competitively sequesters the coactivator CBP from NF-κB and simultaneously induces the transcription of the anti-inflammatory cytokine IL-10, thereby exerting a multi-faceted suppression of NF-κB activity (Biagioli et al., 2017; Högenauer et al., 2014; Haselow et al., 2013).

Beyond its role in suppressing NF-κB signaling, TGR5 is critically involved in regulating the NLRP3 inflammasome (Figure 1). Both *in vivo* and *in vitro* studies have demonstrated that TGR5 activation induces NLRP3 phosphorylation via the cAMP-PKA pathway, which promotes its ubiquitination and subsequent degradation, thereby blocking NLRP3 inflammasomes-dependent inflammation and metabolic disorders (Guo et al., 2016; Iracheta-Vellve et al., 2018; Chen et al., 2019). This protective role is further supported by a model of acute cholestatic liver injury, wherein TGR5-knockdown KCs exhibited accelerated M1 polarization upon LPS stimulation, exacerbating the injury induced by bile duct ligation (BDL). Importantly, inhibition of the NLRP3-ASC inflammasome reversed this effect, confirming that TGR5-mediated protection operates through suppression of the NLRP3 inflammasome (Zhuang et al., 2024). In contrast to this established inhibitory role, one study reported that CDCA induced NLRP3 inflammasome activation and IL-1β secretion in macrophages via a TGR5/EGFR pathway (Gong et al., 2016). However, the physiological relevance of this finding is questionable. First, the study employed a CDCA concentration of 100 μM, which far exceeds the physiological range of BAs in the portal vein (10–80 μM) and peripheral circulation (2–10 μM) (Guo et al., 2016). Second CDCA is a weak agonist for the TGR5 receptor and BAs were revealed to activate the NLRP3 inflammasome via phospho-SP1 under non-inflammatory conditions (Liao et al., 2022), suggesting that the pro-inflammatory effect observed may not be specifically attributable to TGR5/EGFR signaling.

## 3 TGR5 and liver diseases

TGR5 is a major regulator in the liver microenvironment, mediating cell-type-specific responses (Table 2). Research indicates its protective role against hepatic pathologies, which is attributed to anti-inflammatory, anti-steatotic, anti-fibrotic, and cholestasis-alleviating mechanisms. The following sections examine the involvement of this signaling axis in specific diseases, including MASLD, liver fibrosis, cholestatic liver disorders, and HCC.

**TABLE 2:** The regulatory role of TGR5 across different cell types in the iver.

Cell type^a^	Regulation effects
Hepatocytes	Improving glucose tolerance (Holter et al., 2020); promoting hepatocyte apoptosis (Yang et al., 2007); promoting lipotoxicity-induced cell death and inflammation in hepatocytes (Lian et al., 2025)
Cholangiocytes	Inhibiting proliferation of ciliated cholangiocytes but promoting proliferation of nonciliated cells (Masyuk et al., 2013); promoting bile flow and the formation of the bicarbonate-umbrella (Keitel et al., 2009; Reich et al., 2017); inhibiting inflammation (Yao et al., 2023; Di Giorgio et al., 2025); improving biliary epithelial barrier function (Merlen et al., 2020)
Hepatic stellate cells (HSCs)	Inhibiting HSC activation (Sun et al., 2024); promoting HSC apoptosis (Shao et al., 2022); promoting proliferation and activation of HSCs (Xie et al., 2021)
Sinusoidal endothelial cells	Inducing phosphorylation of endothelial nitric oxide synthase and subsequent generation of nitric oxide (Keitel et al., 2007)
Hepatic stem cells	Regulation hepatic differentiation of hepatic stem cells (Sawitza et al., 2015)

^a^ TGR5 regulatory effects on macrophage (KCs) and NKT cells are listed in Table 1.

### 3.1 TGR5 and MASLD

Metabolic dysfunction-associated steatotic liver disease (MASLD), previously termed non-alcoholic fatty liver disease (NAFLD), is a complex metabolic disorder characterized by hepatic lipid accumulation, inflammation, and fibrosis (He et al., 2025). This pathogenesis is driven by dysregulation of glucose, lipid, and cholesterol metabolism (Marroncini et al., 2024). Due to its protective role in metabolic and inflammatory processes, TGR5 has been widely researched in MASLD (Wei et al., 2024). A central piece of evidence for its protective function is the observed decrease in hepatic TGR5 expression in both MASLD patients and experimental models, coupled with the exacerbation of pathology in TGR5^−/−^ mice (Shi et al., 2021). This protective role is further demonstrated by studies showing that TGR5 activation by BAs, synthetic agonists, or natural compounds can alleviate hepatic steatosis, inflammation, and disease progression (He et al., 2024; Yang et al., 2019; Li et al., 2024a; Gillard et al., 2022b; Gillard et al., 2022a).

TGR5 has been proven to regulate blood glucose levels in multiple ways. A well-established pathway involves TGR5-mediated induction of intestinal glucagon-like peptide-1 (GLP-1) release, which improves pancreatic and hepatic function and enhances glucose tolerance, as confirmed by pharmacological and genetic studies (Thomas et al., 2009). Consequently, TGR5-deficient male mice on a high-fat diet exhibit exacerbated insulin resistance compared to their wild-type littermates (Vassileva et al., 2010). This preclinical evidence is supported by clinical research, in which curcumin was found to improve MASLD parameters by modulating gut microbiota-dependent BA metabolism and activating TGR5, thereby reducing fasting blood glucose, triglycerides, and insulin levels (He et al., 2024). Beyond peripheral actions, TGR5 activation also contributes to the regulation of glucose homeostasis within the hypothalamus (Zizzari et al., 2025). Additionally, TGR5 activation has been shown to lower plasma free fatty acids, hepatic triglycerides, and cholesterol levels in various MASLD-related models (Thomas et al., 2009; Finn et al., 2019). In energy metabolism, TGR5 signaling in brown adipose tissue—via the TGR5-cAMP-type 2 iodothyronine deiodinase (D2) pathway—promotes energy expenditure and protects against obesity (Watanabe et al., 2006). TGR5 also upregulates mitochondrial uncoupling protein expression and stimulates hormone-sensitive lipase phosphorylation in adipose tissue, conferring metabolic improvements in diet-induced obese mice (Ding et al., 2021).

Beyond its metabolic actions, TGR5 also counteracts MASLD through its anti-inflammatory properties, targeting the chronic inflammatory signaling that promotes hepatic insulin resistance in obesity (Cai et al., 2005). In regulating inflammation and metabolism, TGR5 and FXR often exhibit synergistic effects in the liver (Häussinger and Keitel, 2018). FXR activation improves metabolic homeostasis primarily through two mechanisms. On one hand, it lowers blood glucose by inhibiting gluconeogenesis and promoting glycogen storage, processes mediated by fibroblast growth factor 19/15. On the other hand, it reduces liver fat by suppressing lipogenesis, which occurs through the small heterodimer partner-mediated inhibition of transcription factors like sterol regulatory element-binding protein 1c (Abdel-Rasol and El-Sayed, 2025). Furthermore, FXR was also reported to directly bind the promoter region of NFKB1 to inactivate NF-κB signaling and suppress inflammation (Jing et al., 2026). Consequently, research interest is growing in dual FXR/TGR5 agonists as a therapeutic strategy for MASLD, with several candidates demonstrating promising efficacy (Zhang et al., 2025; Comeglio et al., 2018; Marchianò et al., 2023). Indeed, simultaneous activation of both receptors is increasingly viewed as a more potent approach than single-receptor targeting for managing metabolic diseases (Pathak et al., 2017; Jadhav et al., 2018; Roth et al., 2018). However, this field contains conflicting findings. For instance, Wang X et al. reported that the dual agonist INT-767 ameliorated western diet–induced MASLD progression exclusively through FXR-dependent mechanisms, with its effects persisting in TGR5 knockout mice but being abolished in FXR knockout mice (Wang et al., 2022). This apparent discrepancy may arise from the unbalanced potency of INT-767, which is a potent FXR agonist (EC_50_ = 30 nM) but a much weaker TGR5 agonist (EC_50_ = 630 nM), resulting in a 21-fold selectivity bias (Rizzo et al., 2010). This underscores the importance of evaluating next-generation, balanced dual agonists (e.g., BAR502) as potential therapies for MASLD. Furthermore, the cell-specific effects of TGR5 activation add another layer of complexity. A recent study revealed that while TGR5 activation in macrophages protects against MASLD progression, its activation in hepatocytes may conversely promote the transition to MASH (Lian et al., 2025). Therefore, future drug development must consider not only receptor agonistic efficacy but also tissue- and cell-type-specific targeting of TGR5.

### 3.2 TGR5 and cholestatic diseases

Cholestatic liver diseases, characterized by impaired bile flow and the accumulation of cytotoxic BAs in the liver, lead to progressive hepatobiliary injury, inflammation, and fibrosis. TGR5, a receptor pivotal in these processes, is abundantly expressed in bile duct cells, as evidenced by its co-localization with the cholangiocyte marker CK-19 (CK19, cholangiocyte marker) (Keitel et al., 2008), and is also present in gallbladder epithelial and smooth muscle cells (Li et al., 2011; Lavoie et al., 2010). Its activation in cholangiocytes exerts multiple protective effects: it stimulates chloride secretion and subsequent bicarbonate-rich choleresis, which mitigates the toxicity of BAs (Guo et al., 2024; Yu et al., 2023), and it regulates the biliary epithelial barrier by modulating JAM-A expression and phosphorylation, thereby preventing bile leakage (Merlen et al., 2020). Furthermore, TGR5 exhibits a complex, cell type-specific role in cholangiocyte proliferation, promoting it in nonciliated cells while inhibiting it in ciliated cells (Masyuk et al., 2013). Given its integral role in governing cholangiocyte secretion, barrier function, and proliferation, TGR5 is a significant player in cholestasis-related diseases.

Beyond its direct effects on cholangiocytes, TGR5 modulates cholestatic liver injury by exerting potent anti-inflammatory effects, primarily through the inhibition of the NF-κB and NLRP3 signaling pathways (Yao et al., 2023; Zhuang et al., 2024). The cell-specific role of TGR5 is complex. Single-cell sequencing revealed that its expression is predominantly high in immune cells, such as macrophages and NKT cells, but relatively low in cholangiocytes. This explains why the overall hepatic TGR5 level can be elevated in primary sclerosing cholangitis (PSC) patients due to immune cell infiltration, despite a disease-associated downregulation of TGR5 specifically within the inflamed biliary epithelium (Di Giorgio et al., 2025; Reich et al., 2021). The functional importance of TGR5 is underscored by *in vivo* evidence: TGR5^−/−^ mice develop more severe liver injury, inflammation, and fibrosis (Rao et al., 2020), whereas activation of TGR5 by specific agonists (e.g., BAR501) or natural compounds (e.g., total astragalus saponins) effectively attenuates cholestatic injury in mouse models of PSC (Di Giorgio et al., 2025; Zhou et al., 2024). Although TGR5 shows promising therapeutic effects for cholestatic diseases, its broad expression characteristics have led to many significant side effects that cannot be ignored. Systemic TGR5 activation has been also reported to worsen pruritus in cholestasis (Alemi et al., 2013; Abu-Hayyeh et al., 2016). The activation of TGR5 in gallbladder smooth muscle cells causes smooth muscle relaxation and promotes gallbladder filling (Lavoie et al., 2010; Li et al., 2011), thereby increasing the risk of cholecystitis and cholelithiasis. These unwanted side effects have become an obstacle to the application of TGR5 agonists (Han et al., 2020).

### 3.3 TGR5 and liver fibrosis

Liver fibrosis, a shared pathological outcome of chronic liver diseases, is characterized by excessive extracellular matrix (ECM) deposition, with HSC activation serving as its central driver. The role of the bile acid receptor TGR5 in this process is complex and exhibits distinct cell-type specificity. Several studies report on a protective role for TGR5. For instance, TGR5 has been shown to suppress HSC activation by inducing apoptosis and inhibiting both Smad-dependent and Smad-independent TGF-β signaling pathways (Shao et al., 2022). Similarly, global TGR5 knockout was found to exacerbate liver fibrosis, and its activation was shown to inhibit HSC activation by attenuating mitochondrial fission, further supporting an overall protective function (Sun et al., 2024). In contrast, other evidence points to a cell-autonomous, pro-fibrotic role within HSCs. Specifically, HSC-specific knockdown of TGR5 was shown to attenuate fibrotic progression (Xie et al., 2021). Mechanistically, in HSCs, TGR5 activation promote the proliferation and activation of HSCs via ERK1/2 and p38 MAPK pathways (Xie et al., 2021). This apparent discrepancy may stem from fundamental differences in experimental models. The studies suggesting a protective role employed global TGR5 knockout, which reflects the net effect of TGR5 loss across all cell types. Conversely, HSC-specific knockdown isolates the function of TGR5 within HSCs themselves, revealing a direct pro-fibrotic signaling role. Thus, TGR5 appears to exert an overall protective role in liver fibrosis at the organismal level, while simultaneously driving a cell-autonomous, pro-fibrotic program within activated HSCs. This dual role highlights the context-dependent nature of TGR5 signaling, though further studies are needed to fully elucidate the conditions that dictate its functional outcome.

The involvement of TGR5 in liver fibrosis extends beyond HSCs to encompass a multicellular regulatory network, wherein it primarily exerts anti-inflammatory and vasomodulatory effects. On one hand, in macrophages, TGR5 activation promotes a metabolic switch from glycolysis to oxidative phosphorylation via the ATP-citrate lyase/Akt pathway, thereby favoring the anti-inflammatory M2 phenotype (Shao et al., 2022). On the other hand, in, LSECs, TGR5 activation counteracts endothelial dysfunction in cirrhotic portal hypertension. It upregulates the expression and activity of cystathionine-γ-lyase (CSE) and endothelial nitric oxide synthase (eNOS) in LSECs, while suppressing endothelin-1 (ET-1) expression via an Akt-dependent FOXO1 phosphorylation mechanism wherein Akt phosphorylates and inactivates the transcription factor FOXO1, which otherwise promotes ET-1 transcription (Renga et al., 2015b; Fiorucci and Distrutti, 2016; Renga et al., 2015a). This restores the balance between vasodilatory and vasoconstrictive forces.

In summary, although TGR5 may exert a direct pro-fibrotic effect within HSCs, its potent anti-inflammatory actions on immune cells (Marchianò et al., 2025; Shao et al., 2022; Di Giorgio et al., 2025) and its vasoprotective benefits on LSECs collectively result in a net anti-fibrotic outcome. The systemic effect likely involves intercellular communication, such as anti-inflammatory cytokines from macrophages (e.g., IL-10) further suppressing HSC activation, and improved LSEC function helping to deactivate HSCs through a healthy paracrine environment, Therefore, systemic TGR5 activation represents a promising therapeutic strategy for liver fibrosis and associated complications, though its cell-specific effects warrant careful evaluation.

### 3.4 TGR5 and HCC

HCC, a highly invasive cancer arising from chronic liver disease and cirrhosis, represents a leading cause of cancer-related mortality worldwide (Wu et al., 2021). The BA receptor TGR5, which regulates energy homeostasis, glucose metabolism, and immunomodulation via cAMP, AKT, and NF-κB signaling pathways, is implicated in hepatic oncogenesis (Li et al., 2019; Qi et al., 2022). Accumulating clinical and mechanistic evidence suggests a tumor-promoting role for TGR5 in HCC. The methylation level of the circulating TGR5 promoter is significantly elevated in patients with HBV-related HCC, indicating its close association with the disease. Furthermore, TGR5 expression is significantly higher in HCC tissues compared to cirrhotic or normal liver tissues, and this upregulation is correlated with bone metastasis and poor patient prognosis (Chen et al., 2023) Mechanistically, the oncogene α7-nicotinic acetylcholine receptor (α7-nAChR) promotes HCC recurrence by upregulating TGR5 and activating the TGR5/JAK2/STAT3 signaling axis, subsequently enhancing the expression of RhoA, ROCK1, MMP2, and MMP9 proteins that facilitate cancer invasion and metastasis (Li et al., 2019). Despite these findings, the role of TGR5 in liver cancer remains complex. While TGR5 has been reported to exert anti-cancer effects in other malignancies (Guo et al., 2015; Su et al., 2017), such a tumor-suppressive function is scarcely documented in HCC, with one supporting study having been retracted. In contrast to the HCC-promoting evidence, numerous studies have unequivocally established the protective roles of TGR5 in HCC precancerous lesions (such as inflammation, metabolic dysfunction, cholestasis, and fibrosis). This raises a pivotal scientific question: following the malignant transformation of hepatocytes, does the function of TGR5 undergo a “switch” from protective to promotive, or is its protective signaling “hijacked” or masked by oncogenic pathways? Currently, precise and systematic investigations into TGR5 across different stages of hepatocarcinogenesis, particularly during the evolution from precancerous lesions to invasive cancer, remain limited. Future research should employ stage-specific genetically engineered animal models, cell-type-specific gene knockout techniques, and longitudinal clinical cohort analyses to elucidate the detailed role of TGR5 in the dynamic progression of HCC and to evaluate its potential as a stage-specific therapeutic target.

## 4 The challenges and opportunities of TGR5-targeted therapy

The TGR5 receptor is ubiquitously expressed across various tissues and cell types, where it modulates processes such as inflammation, energy homeostasis, and metabolism, playing a critical role in pathophysiological mechanisms. The therapeutic targeting of TGR5 represents a promising avenue for the treatment of related diseases. Various TGR5 agonists including BA analogs, natural compounds, and synthetic small molecules have been developed and applied in preclinical studies (Jin et al., 2024; Bhimanwar et al., 2024; Darmanto et al., 2025). These agonists demonstrate great therapeutic potential, but the currently available TGR5 agonists for clinical trials are very limited. GlaxoSmithKline initiated the first clinical trial to evaluate its TGR5 agonist, SB-756050, in individuals with type 2 diabetes (NCT00733577) over 10 years ago. Although SB-756050 showed well-tolerated in subjects, it demonstrated highly variable pharmacodynamic effects both within dose groups and between doses, with increases in glucose seen at the two lowest doses and no reduction in glucose seen at the two highest doses (Hodge et al., 2013). This disappointing result might be due to the properties of SB-756050 or more generally to regional effects of TGR5 activation in the gut. In addition, the widespread expression of TGR5 in the body leads to side effects such as inhibition of gallbladder emptying, pruritus, and reduction in systemic vascular resistance, which consequently limits drug development targeting this pathway (Jin et al., 2024).

Tissue-specific TGR5 agonists have emerged as a new direction in drug development, with gut-targeting demonstrating promise. Activation of TGR5 in intestinal secretory cells stimulates the release of GLP-1 and peptide YY (PYY) (Ullmer et al., 2013; Bala et al., 2014). GLP-1 enhances glucose-dependent insulin secretion, suppresses appetite, and delays gastric emptying, thereby contributing to glycemic control (Gupta et al., 2025). PYY further inhibits appetite (Nguyen and Park, 2025). Consequently, gut-restricted TGR5 agonists hold therapeutic potential for diabetes, obesity, MASLD, and other metabolic disorders (Gou et al., 2023). Gut-restricted drugs require low intestinal permeability to reduce systemic exposure, which can be achieved by increasing the molecular weight or introducing more polar groups. PEGylation modification or introducing a large polar group of N-methylglucamine to TGR5 agonists have achieved certain tissue-specific targeting effects in this regard (Jin et al., 2024). While more clinical studies are needed to verify the safety and efficacy of these agonists in clinical applications.

## 5 Conclusion and future perspectives

The BA membrane receptor TGR5 plays a central role in liver physiology and pathology, coordinating a spectrum of biological functions. Its activation in immune cells mitigates hepatic inflammation by suppressing pro-inflammatory cytokine release via pathways such as NF-κB and the NLRP3 inflammasome. Beyond its anti-inflammatory role, TGR5 is critical in maintaining metabolic homeostasis, improving energy expenditure, insulin sensitivity, and lipid metabolism, which highlights its therapeutic potential for MASLD. Furthermore, TGR5 activation protects against cholestatic injury by modulating cholangiocyte proliferation, secretion, and barrier function. Cell-specific actions also contribute to its hepatoprotective effects: in LSECs, it promotes vasodilation and microcirculation via eNOS activation, while in HSCs, it directly counteracts fibrotic activation.

Despite the broad therapeutic potential of TGR5 activation for chronic liver diseases, its clinical translation faces challenges. Systemic activation causes adverse effects, including gallbladder filling, pruritus, and reduction in systemic vascular resistance, prompting the need for tissue-specific agonists or liver-targeted delivery systems to improve safety. Furthermore, the dynamic role of TGR5 across disease stages remains unclear, necessitating the use of advanced models and single-cell technologies to define its cell-specific functions and optimal intervention windows. Finally, the multifactorial nature of conditions like MASH demands multi-target approaches, such as developing TGR5/FXR dual agonists or combining TGR5 agonists with agents like GLP-1 receptor agonists or FGF19 analogues, to achieve synergistic efficacy. While current compelling evidence for TGR5 primarily comes from animal models, future work must leverage human samples, organoids, and real-world data to verify its mechanistic role and therapeutic value in human pathologies, thus bridging the translational gap. Overall, TGR5 continues to be a highly promising target for liver protection. Addressing the prevailing challenges through continued research and technological innovation is expected to translate TGR5-directed strategies into novel and effective treatments for chronic liver diseases soon.
